# Academic Primer Series: Key Papers About Peer Review

**DOI:** 10.5811/westjem.2017.2.33430

**Published:** 2017-04-19

**Authors:** Lalena M. Yarris, Michael Gottlieb, Kevin Scott, Christopher Sampson, Emily Rose, Teresa M. Chan, Jonathan Ilgen

**Affiliations:** * Oregon Health & Science University, Department of Emergency Medicine, Portland, Oregon; † Rush University Medical Center, Department of Emergency Medicine, Chicago, Illinois; ‡ Perelman School of Medicine at the University of Pennsylvania, Department of Emergency Medicine, Philadelphia, Pennsylvania; § University of Missouri, Columbia, Department of Emergency Medicine, Columbia, Missouri; ¶ Keck School of Medicine of the University of Southern California, Department of Emergency Medicine, Los Angeles, California; || Los Angeles County + USC Medical Center, Department of Emergency Medicine, Los Angeles, California; # McMaster University, Department of Medicine, Division of Emergency Medicine, Hamilton, Ontario, Canada; ** University of Washington School of Medicine, Department of Medicine, Division of Emergency Medicine, Seattle, Washington

## Abstract

**Introduction:**

Peer review, a cornerstone of academia, promotes rigor and relevance in scientific publishing. As educators are encouraged to adopt a more scholarly approach to medical education, peer review is becoming increasingly important. Junior educators both receive the reviews of their peers, and are also asked to participate as reviewers themselves. As such, it is imperative for junior clinician educators to be well-versed in the art of peer reviewing their colleagues’ work. In this article, our goal was to identify and summarize key papers that may be helpful for faculty members interested in learning more about the peer-review process and how to improve their reviewing skills.

**Methods:**

The online discussions of the 2016–17 Academic Life in Emergency Medicine (ALiEM) Faculty Incubator program included a robust discussion about peer review, which highlighted a number of papers on that topic. We sought to augment this list with further suggestions by guest experts and by an open call on Twitter for other important papers. Via this process, we created a list of 24 total papers on the topic of peer review. After gathering these papers, our authorship group engaged in a consensus-building process incorporating Delphi methods to identify the papers that best described peer review, and also highlighted important tips for new reviewers.

**Results:**

We found and reviewed 24 papers. In our results section, we present our authorship group’s top five most highly rated papers on the topic of peer review. We also summarize these papers with respect to their relevance to junior faculty members and to faculty developers.

**Conclusion:**

We present five key papers on peer review that can be used for faculty development for novice writers and reviewers. These papers represent a mix of foundational and explanatory papers that may provide some basis from which junior faculty members might build upon as they both undergo the peer-review process and act as reviewers in turn.

## INTRODUCTION

Peer review is a key component of academic publishing, and aims to provide rigor and relevance to the publishing process.^1^ While the primary aim of the peer-review process is to select and prepare manuscripts for publication, the service of peer review also provides professional development, reward, and opportunities for further scholarship to the reviewer. However, faculty new to peer review may feel intimidated or unprepared to engage in this scholarly activity.

While peer review draws upon skills that many faculty already have, it does require content and process knowledge that is rarely formally taught to novice reviewers. Quality peer review does not necessarily correlate with traditional markers of experience, such as academic rank, research training, or grant funding.^2^ While peer review has traditionally been a solitary practice, models are emerging that facilitate a mentored or team-based approach. These approaches allow junior faculty to receive mentorship in the one-to-one mentored model, and engage in a community of practice in a team-based approach.^3^,^4^ However, a foundational understanding of the elements of a quality peer review and the peer-review process can be helpful prior to engaging in peer review.

The Faculty Incubator was created by the Academic Life in Emergency Medicine (ALiEM) team to provide early-career educators with a community of practice where they can discuss and debate topics relevant to the 21st century medical educator. To that end, we created a one-month module focused on peer review.

This paper is a narrative review that highlights some important literature that may assist junior educators who are seeking to learn more about the peer-review process.

## METHODS

In the seventh month of the ALiEM Faculty Incubator (September 2016), we discussed the topic of peer review. We monitored the proceedings of this group of educators from September 1–30, 2016. Our online discussions involved both junior faculty members and faculty mentors. While discussions occurred, we gathered the titles of papers that were cited, shared and recommended within our online discussion forum and compiled these into a list.

This list was then augmented by the following: 1) A YouTube Live session with experts Drs. Jonathan Ilgen & Lalena Yarris, who are both medical educators and editors at *Academic Emergency Medicine Education & Training* and the *Journal of Graduate Medical Education*; 2) A YouTube Live session with Drs. Ellen Weber & Michael Callaham who are both editors of leading EM journals; and 3) a call for important papers regarding peer review on Twitter. We “tweeted” requests to have participants of the #PeerReview, #FOAMed and #MedEd online communities provide suggestions for important papers on the topic of peer review. Figure demonstrates an exemplar tweet.

Once the augmented list was completed, we then conducted a three-round voting process, similar to our previously described Delphi-inspired method to build consensus around which papers to feature.^5^ This was a modified Delphi method since our selection panel was comprised of both novices (i.e., junior faculty members, participants in the Faculty Incubator) and experts in the field (i.e., experienced clinician educators, all of whom have published >10 peer-reviewed publications, who serve as mentors and facilitators of the ALiEM Faculty Incubator). However, we intentionally used this method so as to involve both junior and experienced clinician educators to ensure we selected papers that would be of use to a spectrum of educators throughout their careers.

## RESULTS

Our ALiEM Faculty Incubator discussions in combination with expert recommendations and social media calls yielded a total of 24 articles. Our procedure allowed us to create a rank-order listing of all these papers in order of perceived relevance, from the most to the least relevant. The top five papers were expanded upon below. Our ratings of all 24 papers are listed in the Table, along with their full citations.

## DISCUSSION

Our group determined the following papers to be of highest interest and relevance to novice reviewers and faculty developers. The accompanying commentaries are meant to explain the relevance of these papers to junior faculty members, and also highlight considerations for senior faculty members when using these works for faculty development workshops or sessions.

### 1. Lovejoy TI, Revenson TA, France CR. Reviewing manuscripts for peer-review journals: a primer for novice and seasoned reviewers. *Ann Behav Med*. 2011 Aug 1;42(1):1–3.^1^

#### Summary

Lovejoy and colleagues provide an overview of the peer-review process for *Annals of Behavioral Medicine*, describing and providing examples of high-quality reviews for that journal. Although the focus of the process is specific to the behavioral and social-science focus of this journal, the general principles are largely applicable to most academic journals and fields. Specifically, the authors raise awareness of the need for more formal reviewer guidance and attempt to do so by way of this manuscript.

The authors begin by discussing the roles of the editors and editorial board for the journal and laying out the responsibilities of each. Of special interest to potential reviewers is the process by which action editors select reviewers, highlighting the importance for new reviewers to only identify actual areas of personal expertise. The majority of the article focuses on the actual process of reading a manuscript and drafting the review, including specific considerations pertaining to each of the separate sections of a manuscript. The authors provide a framework for critically appraising manuscripts by explicitly highlighting the roles of the reviewer in order to 1) offer opinions on the strengths and weaknesses of a manuscript, and 2) provide guidance to authors in how to improve scientific process and communication. To conclude, the authors summarize the “do’s and don’ts” of the peer-review process in addition to providing an annotated example of a high-quality review from a paper published previously within the journal.

#### Relevance to Junior Faculty Members

This paper is relevant to junior faculty who wish to participate in peer review for service, personal professional development, and as a scholarly activity for career advancement. The paper provides an understanding of the peer-review process that is crucial to being able to perform the responsibilities of a reviewer. Although not the focus of the article, a common mistake made by novice peer reviewers is overextending themselves: This may include attempting to review beyond the limits of their actual expertise. A better approach is to select fewer areas of expertise in order to build a portfolio of timely, high-quality reviews, expanding knowledge with progression of one’s career, leading to future review opportunities.

The largest area of relevance for junior faculty in this article is found in the step-by-step approach to performing a review. The authors provide a guide for reviewers, starting with accepting or declining an invitation to review and concluding with review submission. Additionally, reviewers should consider reading the articles at least once without marking or making comments, just to assess for readability and understanding.

This article then provides a concise yet complete series of considerations for each section of a manuscript, which can help guide the novice reviewer’s thought process and ultimately drafting of the review. Additionally, the article provides two different options for organizing the review, highlighting the necessity to identify major versus minor concerns. Novice reviewers may find this article a useful guide, providing a framework for initial reviews that will likely become more intuitive with experience and time.

#### Considerations for Faculty Developers

This paper provides a useful “how-to” resource for faculty developers to prepare academic faculty for peer review. It is a broad and comprehensive overview that provides both step-by-step instructions on the process, and examples to highlight how to apply these instructions to an actual review.

### 2. Azer SA, Ramani S, Peterson R. Becoming a peer reviewer to medical education journals. *Med Teach*. 2012;34(9):698–704.^6^

#### Summary

As part of the *Twelve Tips* series, this paper provides valuable advice for the more novice peer reviewer. The authors discuss the importance of gauging your qualifications, any significant biases, and available time prior to agreeing to review (Tips 1, 3, 5, and 6). They also emphasize the role of the reviewer, not only in critically appraising the article itself, but also in determining how well the submission fits within the journal’s style and mission (Tips 2 and 4). They address the importance of confidentiality and professionalism, highlighting the need to keep critiques constructive and reminding the reviewer that the purpose is to strengthen the paper (Tips 7, 8, and 9). The last three tips are, perhaps, the most valuable of all. Tip 10 addresses confidential comments to the editor, clarifying what should be included and the importance of consistency between these recommendations and those shared with the authors. Tip 11 emphasizes the differences between educational and basic scientific research, reminding those reviewing in education journals the differences in approaches and limitations. Finally, tip 12 provides a variety of strategies to improve one’s peer-review skills. While isolated interventions have not significantly influenced peer-review skills,^7^–^10^ using this combination of strategies may be more fruitful.

#### Relevance to Junior Faculty Members

As junior faculty members become involved in peer review, it is important to keep some core components in mind. This paper provides a concise table highlighting key questions for each component of the submission. The table in this paper can serve as a simple one-page guide for the more novice reviewer to help structure his/her reviews. The subsequent tips emphasize some of the less tangible, but equally important, components of the review process. From a professionalism standpoint, this paper reminds the potential reviewer that s/he should ensure that s/he is adequately qualified and unbiased, and able to provide constructive criticism, rather than simply highlighting faults. The paper also highlights the importance of providing an overview, general recommendations, and assessment of suitability for the journal in addition to the discussion of specific suggestions. Finally, the paper highlights numerous strategies for improving one’s peer-review skills. Examples include attending peer-review workshops, reading papers highlighting strategies for producing high quality reviews, reading other reviewers’ comments from the same paper, asking for feedback from the editor and colleagues, and reflection on one’s experiences.

#### Considerations for Faculty Developers

This paper provides a helpful roadmap to guide and orient novice reviewers to the many steps and factors impacting the peer-review process, and many of these are concisely summarized in the Table. Tips 2 (“Familiarize yourself with the journal style”) and 11 (“Know the differences between educational and scientific research”) highlight the value of mentorship for novice reviewers, as these subtle differences in article types may not be immediately apparent to those who are less familiar with the medical education literature, and reviewers may feel ill-prepared to critique research approaches that fall outside of their more traditional biomedical training. Guiding novice reviewers to be introspective about both potential conflicts of interest (Tip 3) and bias (Tip 6) are essential mindsets, and allowing time for reflection (Tip 5) will encourage reviewers to provide the most thoughtful and nuanced suggestions for improvement.

### 3. Bordage G. Reasons reviewers reject and accept manuscripts: the strengths and weaknesses in medical education reports. *Acad Med*. 2001 Sep;76(9):889–96.^11^

#### Summary

This study sought to explore the strengths and weaknesses of submissions after analyzing peer-reviewer ratings and comments. A content analysis of the 151 peer-reviewed research manuscripts submitted to the 1997 and 1998 Association of American Medical College- sponsored *Research in Medical Education* (RIME) conference was performed. Peer reviewers for RIME come from around the world, and all accepted manuscripts are published in a supplement of *Academic Medicine.* Each masked submission was evaluated by four or five reviewers who work as medical educators. Anonymous comments and a review form are completed by each reviewer. Eight areas are rated on a five-point scale (excellent, good, fair, unsatisfactory and not acceptable). The eight areas rated are problem statement and background, research design, sampling, instrumentation and data collection, results, conclusion, writing and importance. Finally, each reviewer is asked to use a four-point (definitely include; acceptable, probably include; questionable, probably exclude; definitely exclude) global rating and give additional comments on merits or shortcomings of submission.

Interestingly, nearly two fifths of the reviewers recommended rejection without any unsatisfactory ratings on the checklist. The top reason for rejection was inappropriate, incomplete or insufficiently described statistics. This was followed by over-interpretation of results. The top reason for manuscript acceptance was importance, timeliness, relevance, and critical pertinent problem. Both good and bad quality of writing was raised by many reviewers, stressing the importance of well-written manuscripts. Acknowledging limitations rather than ignoring them was also deemed important. As summarized by Bordage, “scientific writing demands both good science and writing good manuscripts.”

#### Relevance to Junior Faculty Members

Bordage highlights important items that junior faculty should consider when taking part in the peer-review process. The ability of a reviewer to determine what is a well-written manuscript and what are appropriate statistics for a study seem most important. This article implies that peer reviewers should have a background in statistics in order to be able to interpret analysis and results as appropriate. A junior peer reviewer based on this study should also be able to critically appraise a research project for its well-performed design, timeliness and novel approach. The ability of the study to provide practical, useful implications should also be considered. The junior reviewer must also be able to provide written comments and feedback to the authors in order to provide guidance in what can be improved.

#### Considerations for Faculty Developers

This paper is a great launching point for a discussion on how to improve both peer review and quality of writing with junior scientists. By being aware of the common “fatal flaws” encountered in the field of medical education, it is possible to then pay more attention to these problems when reviewing papers. Faculty developers may want to use the lists generated by this paper to create some easy-to-use handouts for guiding junior faculty members when critically appraising their own work as well. Discussions around each of the most common grounds for rejection and acceptance can be used to scaffold journal club proceedings or internal peer-review processes of research units.

### 4. Eva KW. The reviewer is always right: peer review of research in medical education. Med Educ. 2009 Jan;43(1):2–4.^12^

#### Summary

This editorial, written by the editor-in-chief of *Medical Education,* discusses the importance of understanding and incorporating reviewer comments, even when the author does not entirely agree with them. The author highlights the importance of the peer-review process for improving a manuscript, emphasizing the value in both well-written, high-caliber reviews, as well as those in which the reviewer is unclear or incorrect in their interpretation. In the latter case, Eva emphasizes that peer reviewers are reading submissions much more carefully than the standard readership and that any confusion should prompt the author to reevaluate the text and address any ambiguity. He subsequently discusses the importance of peer review and provides several strategies for improvement, which include the provision of a guideline for reviewers, deliberate feedback, and maximizing opportunities to review.

#### Relevance to Junior Faculty Members

After devoting significant time and effort to a publication, junior faculty may become frustrated after receiving reviewer critiques, especially when the reviewer expresses confusion over what appeared so clear to the author. This paper reminds the junior faculty member that reviewer comments are valuable both by emphasizing what may have been missed, as well as those aspects which may be unclear to readers. It is advisable after receiving reviewer comments to set the manuscript aside for several days and return after the emotions have passed and empathize with the reviewer’s comments and perspective. Junior faculty may also benefit by seeking feedback from colleagues and mentors prior to submission. Finally, when serving as a reviewer, junior faculty should review the existing guidelines and seek feedback to ensure that they continually improve their peer-review skills.

#### Considerations for Faculty Developers

This paper highlights several important concepts for faculty teaching peer-review skills to others. In particular, adopting the maxims of “Did I learn anything?” and “What could the authors have done to convince me of the argument they are trying to convey?” frames peer review as an activity rooted in the goal of providing actionable feedback to authors that will help them to improve their work (as opposed to simply giving summary judgments on the manuscript’s overall quality). The *Medical Education* reviewer guidelines (com ) highlighted in this article also provide a useful rubric for the types of general issues that reviewers should consider when conducting a review.

### 5. Lucey B. Peer review: how to get it right—10 tips. *The Guardian*. September 27, 2013.^13^

#### Summary

Lucey succinctly details advice for peer review in plain language. His 10 tips are concise and capture the essentials of peer reviewing. The first tip is to “be professional,” meaning participate in review because it is a professional obligation as well a means to enhance your own writing. Tips two through four are “be pleasant,” “read the invite” [to review] and follow its specific instructions, and “be helpful.” Don’t only identify shortcomings but offer suggestions to fix identified problems. The fifth tip, “be scientific” emphasizes the reviewer’s essential role. The reviewer contribution is expertise in scientific knowledge (not proofreading). Six, “be timely.” Editors will notice when you stick to deadlines (and don’t). Tip seven, “be realistic” about the reviewing role. The reviewer has an important contribution to make, but not the final say in the ultimate decision regarding publication of the paper. Eight, “be empathetic” in the review and treat others the way that you would want to be treated. Tip nine is “be open” to performing a review even if it is not an area of expertise. Generalists (i.e., non-subject specialists) make significant contributions to the readability and practicality of papers. Finally, tip ten is “be organized.” The review is a communication that requires structure and logical flow. Follow the publisher’s recommended review structure (if available). Specifically, start with an overview, give feedback on the paper structure, quality of data sources, investigation methods, methodology, flow of argument, and validity of conclusions. Comment on the paper style/voice and give specific suggestions for improvement.

#### Relevance to Junior Faculty Members

Peer review is an essential part of an academic career and most junior faculty flounder a bit with the first reviews. This article emphasizes the “big picture” of peer review. It is important because it so clearly and simply states the appropriate responsibility and behavior of an excellent and thoughtful reviewer. It provides an easy-to-follow outline of issues that a reviewer must address when evaluating a paper. The most important emphasis of this paper for junior faculty is the advice to not only find flaws in a paper, but help find solutions. This is an essential skill in thinking critically, evaluating scientific literature and in ultimately developing an academic career.

#### Considerations for Faculty Developers

This is a pragmatic article that can be used as a springboard for discussing the role and integration of peer-review responsibilities for those new to the job. Faculty developers will find this a useful guide for reminding faculty members (who may have experienced the slings and arrows of blinded peer review) about how to provide positive and constructive peer reviews. This paper may be a useful prophylaxis against the negative feelings that can emerge between reviewees and reviewers, reminding us to be empathetic, helpful, and kind – rather than unremittingly blunt, mean, or sarcastic.

## LIMITATIONS

As with our previous papers, we did not design this study to be an exhaustive, systematic search of the literature. We attempted to seek assistance with finding more papers by using expert consultation and an open social media call via Twitter using hashtags #MedEd & #FOAMed, which yielded some important recommended papers. Considering the depth and breadth of our final list, we feel that by using these adjunctive methods we have overcome the limitations of our unstructured collection of papers.

## CONCLUSION

We provide a reading list on the topic of peer review that may be beneficial as a primer for junior clinician educators and as a potential reading list for senior faculty members leading faculty development efforts. We hope this paper may serve as a guide for clinician educators who are looking to further the development of their own peer-review skills.

## Figures and Tables

**Figure:**
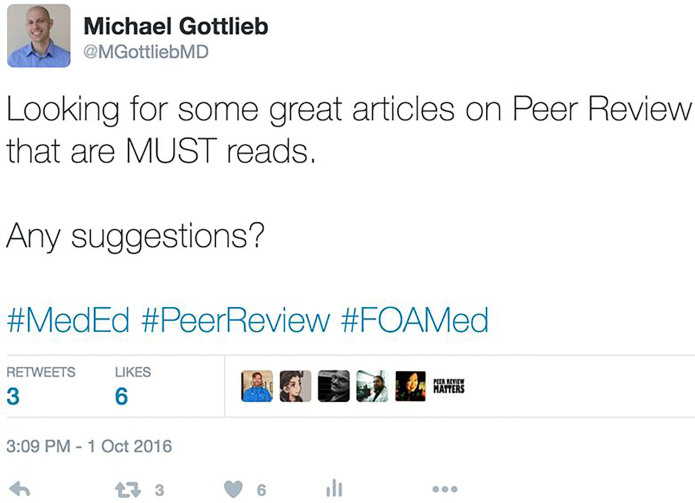
Exemplar tweet soliciting relevant papers on peer review.

**Table:** The complete list of peer-review literature collected by the authorship team.

Citation	Round 1 initial mean scores (SD) max score 7	Round 2 % of raters that endorsed this paper	Round 3 % of raters that endorsed paper in last round	Top 5 papers
Lovejoy TI, Revenson TA, France CR. Reviewing manuscripts for peer-review journals: a primer for novice and seasoned reviewers. *Ann Behav Med*. 2011;42(1):1–3.^1^	6.7 (0.5)	100%	100%	1
Azer SA, Ramani S, Peterson R. Becoming a peer reviewer to medical education journals. *Med Teach*. 2012;34(9):698–704.^6^	6.5 (0.5)	100%	100%	2
Roediger HL III. Twelve tips for reviewers. Observer. April 2007. Available at: http://www.psychologicalscience.org/index.php/pupublicatio/observer/2007/april-07/twelve-tipsfor-reviewers.html. Accessed December 17, 2016.^14^	6.3 (1.0)	100%	28.6%	
DeMaria AN. What constitutes a great review? *J Am Coll Cardiol*. 2003;42(7):1314–5. 15	5.9 (0.9)	86.7%	14.3%	
Eva KW. The reviewer is always right: peer review of research in medical education. *Med Educ*. 2009;43(1):2–4.^12^	5.9 (1.1)	100%	71.4%	4
Lucey B. Peer review: How to get it right—10 tips. The Guardian. September 27, 2013. Available at: http://www.theguardian.com/higher-education-network/blog/2013/sep/27/peer-review-10-tips-research-paper?CMP¼twt_gu. Accessed last December 17, 2016.^13^	5.7 (1.1)	100%	42.9%	5
Dumenco L, Engle DL, Goodell K, et al. Expanding group peer review: a proposal for medical education scholarship. *Acad Med*. 2016 Sep 27. [Epub ahead of print]^4^	5.4 (1.4)	71.4%	28.6%	
Bordage G. Reasons reviewers reject and accept manuscripts: the strengths and weaknesses in medical education reports. *Acad Med*. 2001;76(9):889–96.^11^	5.3 (1.0)	100%	85.7%	3
Shea JA, Caelleigh AS, Panagaro L, et al. Review process and publication decision. *Acad Med*. 2001;76(9):911–21.^16^	5.4 (1.4)	85.7%	28.6%	
Triggle CR, Triggle DJ. What is the future of peer review? Why is there fraud in science? Is plagiarism out of control? Why do scientists do bad things? Is it all a case of: “all that is necessary for the triumph of evil is that good men do nothing”? *Vasc Health Risk Manag*. 2007;3(1):39–53.^17^	4.1 (1.3)	42.9%	0%	
Evans AT, McNutt RA, Fletcher SW, et al. The characteristics of peer reviewers who produce good-quality reviews. *J Gen Intern Med*. 1993;8(8):422–8.^18^	4.9 (1.3)	85.7%	0%	
Thoma B, Chan T, Desouza N, et al. Implementing peer review at an emergency medicine blog: bridging the gap between educators and clinical experts. *CJEM*. 2015;17(2):188–91.^19^	4.6 (0.8)	28.6%	0%	
van Rooyen S, Delamothe T, Evans SJ. Effect on peer review of telling reviewers that their signed reviews might be posted on the web: randomised controlled trial. *BMJ*. 2010;341:c5729.^20^	4.4 (1.5)	42.6%	0%	
Green SM, Callaham ML. Implementation of a journal peer reviewer stratification system based on quality and reliability. *Ann Emerg Med*. 2011;57(2):149–152.e4.^21^	4.3 (1.5)	28.6%	0%	
Sidalak D, Purdy E, Luckett-Gatopoulos S, et al. Coached peer review: developing the next generation of authors. *Acad Med*. 2016 May 17. [Epub ahead of print]^22^	4.1 (1.1)	14.3%	0%	
Cooper LB, Bellam N, Vaduganathan M; JACC: Heart failure fellows. Educating the next generation of peer reviewers. *J Am Coll Cardiol*. 2016;67(17):2079–82.^23^	4.1 (1.7)	0%	0%	
Monrouxe L, Haidet P, Ginsburg S, et al. Good advice from the deputy editors of medical education. *Med Educ*. 2012 Sep;46(9):828–9.^24^	3.7 (1.6)	0%	0%	
Callaham M, McCulloch C. Longitudinal trends in the performance of scientific peer reviewers. *Ann Emerg Med*. 2011;57(2):141–8.^25^	3.7 (1.5)	0%	0%	
Callaham ML, Tercier J. The relationship of previous training and experience of journal peer reviewers to subsequent review quality. *PLoS Med*. 2007;4(1):e40.^26^	3.7 (1.8)	014.3%	0%	
Callaham ML, Knopp RK, Gallagher EJ. Effect of written feedback by editors on quality of reviews: two randomized trials. *JAMA*. 2002;287(21):2781–3.^9^	3.6 (1.0)	0%	0%	
Houry D, Green S, Callaham M. Does mentoring new peer reviewers improve review quality? A randomised trial. *BMC Med Educ*. 2012;12:83.^8^	3.4 (1.0)	0%	0%	
Callaham ML, Schriger DL. Effect of structured workshop training on subsequent performance of journal peer reviewers. *Ann Emerg Med*. 2002;40(3):323–8.^10^	3.3 (1.4)	0%		
Callaham ML, Wears RL, Waeckerle JF. Effect of attendance at a training session on peer reviewer quality and performance. *Ann Emerg Med*. 1998;32(3 Pt 1):318–22.^7^	3.1 (1.2)	0%		
Norman, G. Editorial—How bad is medical education research anyway? *Adv Health Sci Educ Theory Pract*. 2007;12(1):1–5.^27^	2.9 (0.9)	0%		
